# Effects of Attentional Focus on Muscular Endurance: A Meta-Analysis

**DOI:** 10.3390/ijerph19010089

**Published:** 2021-12-22

**Authors:** Jozo Grgic, Pavle Mikulic

**Affiliations:** 1 Institute for Health and Sport, Victoria University, Melbourne 3011, Australia; 2 Faculty of Kinesiology, University of Zagreb, 10000 Zagreb, Croatia; pavle.mikulic@kif.unizg.hr

**Keywords:** motor learning, resistance training, strength endurance

## Abstract

Several studies explored the effects of attentional focus on resistance exercise, but their analysed outcomes most commonly involved surface electromyography variables. Therefore, the effects of attentional focus on resistance exercise performance remain unclear. The aim of this review was to perform a meta-analysis examining the acute effects of external focus vs. internal focus vs. control on muscular endurance. Five databases were searched to find relevant studies. The data were pooled in a random-effects meta-analysis. In the analysis for external vs. internal focus of attention, there were seven comparisons with 14 study groups. In the analyses for external focus vs. control and internal focus vs. control, there were six comparisons with 12 study groups. An external focus of attention enhanced muscular endurance when compared with an internal focus (Cohen’s *d*: 0.58; 95% confidence interval (CI): 0.34 and 0.82) and control (Cohen’s *d*: 0.42; 95% CI: 0.08 and 0.76). In the analysis for internal focus vs. control, there was no significant difference between the conditions (Cohen’s *d*: –0.19; 95% CI: –0.45 and 0.07). Generally, these results remained consistent in the subgroup analyses for upper-body vs. lower-body exercises. From a practical perspective, the results presented in this review suggest that individuals should use an external focus of attention for acute enhancement of muscular endurance.

## 1. Introduction

The effects of attentional focus on motor learning have been explored for over 20 years [1]. In the first study published on this topic, Wulf et al. [2] examined the effects of attentional focus on skiing movements performed on a ski simulator. This study compared the effects of internal focus (focusing on participant’s body movements) vs. external focus (focusing on movements on the apparatus) [2]. The results indicated that adopting an external focus of attention enhanced motor learning [2]. Since then, many studies have examined the effects of attentional focus on various tasks [1,2,3,4,5,6]. Currently, there is agreement that an external focus of attention enhances different aspects of motor performance, such as accuracy, consistency, and balance [1,2,3].

While the effects of attentional strategies have been explored for various exercise tasks, the influence of external vs. internal focus on resistance exercise has received less attention. Several studies explored the effects of attentional focus on resistance exercise [4,5,6]. For example, in the study by Vance et al. [5], participants performed bicep curls with instructions to concentrate on their arms (internal focus) or on the barbell (external focus). This study observed that integrated electromyography (EMG) activity was lower in the external focus condition [5]. Previous studies focused on the EMG-derived outcomes, likely because of the suggested importance of the “mind–muscle” connection in resistance exercise [6]. While the findings presented in these studies are certainly of interest, they do not provide us with insights into the effects of external vs. internal focus on resistance exercise performance. 

Several muscular qualities are important when discussing resistance exercise, including muscular endurance [7]. Kell et al. [7] defined muscular endurance as “the ability of a muscle or muscle group to perform repeated contractions against a load for an extended period.” Several studies examined the influence of external focus of attention on muscular endurance but their observed effects varied [8,9,10,11,12]. Collum et al. [9] explored the effect of external focus using the following instruction “drive the weight towards the ceiling” vs. internal focus using the instruction to “drive the weight with your chest” on performance in the bench press. In this study, the participants performed a greater number of repetitions in the external focus condition. While others also support these findings, it should be considered that the effect size of external focus on muscular endurance ranged from small to very large (Cohen’s *d*: 0.28 vs. 1.28), making it difficult to establish the magnitude of the true effect in the population [10]. Additionally, some studies reported that an external focus improves muscular endurance compared with an internal focus of attention but not a control condition (i.e., no instructions provided) [10]. Due to conflicting reports, there is still no consensus on this topic. 

Several narrative reviews briefly mentioned the effects of attentional focus on muscular endurance and suggested that using an external focus of attention may enhance this muscular quality [1,13,14]. However, these reviews did not contain a meta-analysis that pooled data from available studies. This would be important to perform, given that some of the studies published on the topic might have been underpowered to find significant differences. Therefore, this review aimed to perform a meta-analysis examining the effects of external focus vs. internal focus vs. control on muscular endurance.

## 2. Materials and Methods

### 2.1. Search Strategy

A search through five databases was performed to identify studies that explored the effects of attentional focus on muscular endurance. Specifically, we searched through Networked Digital Library of Theses and Dissertations, PubMed/MEDLINE, Scopus, SPORTDiscus, and Web of Science. The search syntax used in all databases included the following terms and Boolean operators: (“focus of attention” OR “attentional focus” OR “external focus” OR “internal focus”) AND (“strength endurance” OR “muscular endurance” OR “resistance exercise” OR “resistance training” OR “bench press” OR “squat" OR “push-up*”). The search through the databases was performed on 2 October 2021. After completing this phase of the search process, secondary searches were conducted. These included examining (a) the reference lists of the included studies and (b) the papers that cited the included studies using the Google Scholar database.

### 2.2. Inclusion Criteria

To be included in this review, studies were required to satisfy the following criteria:

1. Published in English;
2. Utilized a crossover study design;
3. Examined the effects of external focus vs. internal focus on muscular endurance (with or without a control condition); and
4. Used an isotonic test of muscular endurance, where the outcome was the number of repetitions completed.

### 2.3. Data Extraction

From all included studies, the following data were extracted:

1. Lead author name and the year of study publication;
2. Characteristics of the included participants;
3. Instructions provided to the participants during the experimental trials;
4. Description of the test used to evaluate muscular endurance; and
5. Mean ± standard deviation (SD) muscular endurance data from the experimental trials.

Instead of SD, several studies presented standard errors [10,11,12]. For these studies, standard errors were converted to SDs. As one study presented the mean ± SD in a figure, these data were extracted using the Web Plot Digitizer software.

### 2.4. Methodological Quality

Methodological quality and risk of bias appraisal of the included studies were performed using the 11-item PEDro checklist (https://pedro.org.au/english/resources/pedro-scale/ (accessed on 20 December 2021)) [15]. This checklist is designed to evaluate various aspects of study quality, such as inclusion criteria, randomization, allocation concealment, blinding, attrition, and data reporting. If a criterion was not satisfied, a score of “0” was given. In contrast, if the criterion was satisfied, a score of “1” was given. The maximum number of points that can be scored on the PEDro checklist is 10 because the first item does not contribute to the summary score. Classification of studies is based on the following thresholds:

1. Excellent quality for 9–10 points;
2. Good quality for 6–8 points;
3. Fair quality for 4–5 points; and
4. Poor quality for ≤3 points [16,17].

### 2.5. Statistical Analysis

Meta-analyses were performed using effect sizes (Cohen’s *d*) and the random-effects model. The muscular endurance performance values were converted to Cohen’s *d* using the mean ± SD data from the trials and sample size. Meta-analyses were performed to compare the effects of external focus vs. internal focus, external focus vs. control, and internal focus vs. control. For each of the performed comparisons, subgroup analyses were performed for upper-body vs. lower-body resistance exercises. Cohen’s *d* was interpreted using the following thresholds: trivial (<0.20), small (0.20–0.49), medium (0.50–0.79), and large (≥0.80) [18]. Heterogeneity was evaluated using *I*^2^ and interpreted as low (<50%), moderate (50–75%), and high heterogeneity (>75%). The statistical significance threshold was set at *p* < 0.05. All analyses were performed using the Review Manager software, version 5.4 (Nordic Cochrane Centre, Copenhagen, Denmark; Cochrane Collaboration).

## 3. Results

### 3.1. Search Results

**Figure 1:**
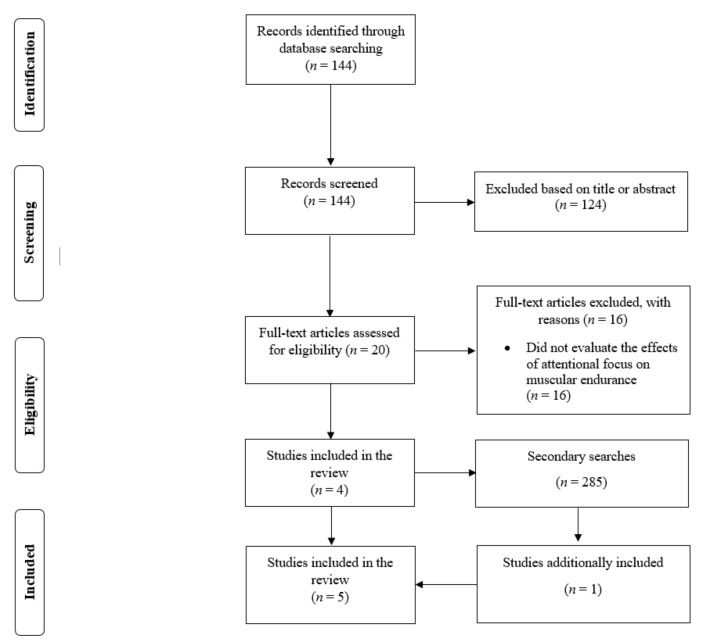
Depiction of the search process.

In the primary search, there were 144 search results. Out of this pool of references, 124 results were excluded based on the title or abstract. Therefore, 20 full-text studies were read and four studies [8,10,11,12] were found to satisfy the inclusion criteria. In the secondary search, there were 285 search results. We excluded 284 based on title, abstract, or full-text, and one study [9] was additionally included. Therefore, a total of five studies were included in the review (Figure 1) [8,9,10,11,12]. One study included male and female participants and explored sex-specific effects [8]. Accordingly, data for males and females were analysed separately. Marchant et al. [10] included two experiments on different participants within the same study, and the data for each experiment were also analysed separately. Therefore, in the analysis for external vs. internal focus, there were seven comparisons with 14 study groups. In the analyses for external focus vs. control and internal focus vs. control, there were six comparisons with 12 study groups.

### 3.2. Summary of Studies

The pooled number of participants among the included studies was 141 (24 females and 117 males). In most of the included studies, the participants were resistance-trained individuals. Studies used different exercises to evaluate muscular endurance, including push-ups, bench press, squat, and deadlift. Four studies first tested their participants’ one-repetition maximum (1RM; using the 1RM test or prediction equations) and then adjusted the load for the muscular endurance test using 75% or 85% of 1RM [9,10,11,12]. One study used an absolute load, where the male and female participants were required to lift 40 kg and 20 kg for the bench press until failure, respectively [10]. Finally, one study used a bodyweight exercise (i.e., push-up) [9]. In all studies, a single set until muscular failure was performed in each testing condition. The specific cues provided to the participants in the external and internal focus conditions are summarized in Table 1.

### 3.3. Methodological Quality

Three studies [8,9,11] scored six points on the PEDro checklist and were classified as being of good methodological quality. Two studies [10,12] scored five points and were classified as being of fair methodological quality.

### 3.4. Meta-Analysis Results

**Figure 2:**
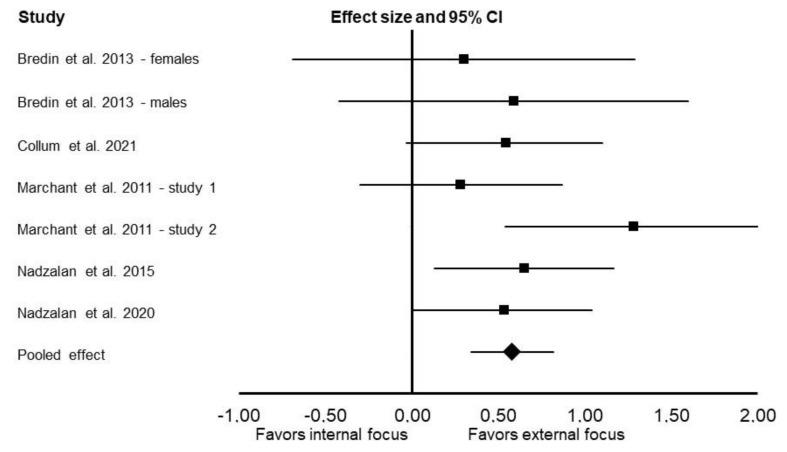
Results of the random-effects meta-analysis comparing the effects of external focus vs. internal focus on muscular endurance. Data are reported as effect sizes (Cohen’s *d*) and 95% confidence interval (CI). The diamond at the bottom presents the overall effect. The plotted squares denote effect sizes, and the whiskers denote their 95% CIs.

Compared with an internal focus, using an external focus of attention enhanced muscular endurance (Cohen’s *d*: 0.58; 95% CI: 0.34 and 0.82; *p* < 0.001; *I*^2^ = 0%; Figure 2). An external focus of attention also enhanced muscular endurance in upper-body exercises (Cohen’s *d*: 0.53; 95% CI: 0.24 and 0.83; *p* = 0.0003; *I*^2^ = 12%) and lower-body exercises (Cohen’s *d*: 1.36; 95% CI: 0.32 and 2.40; *p* = 0.01; *I*^2^ = 87%).

**Figure 3:**
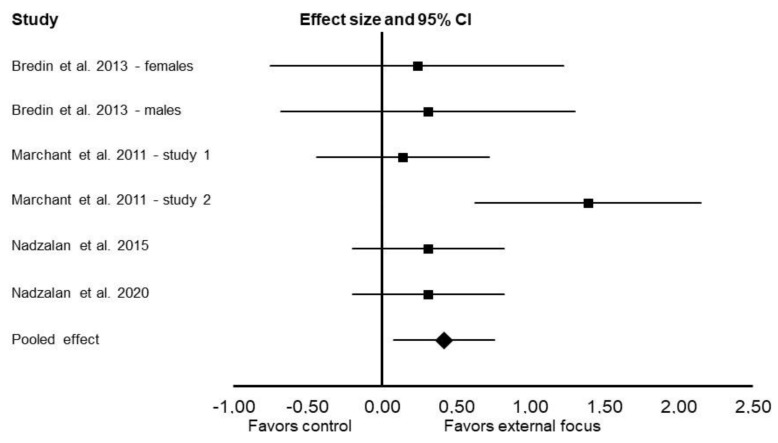
Results of the random-effects meta-analysis comparing the effects of external focus vs. control on muscular endurance. Data are reported as effect sizes (Cohen’s *d*) and 95% confidence interval (CI). The diamond at the bottom presents the overall effect. The plotted squares denote effect sizes, and the whiskers denote their 95% CIs.

Compared with the control, using an external focus of attention enhanced muscular endurance (Cohen’s *d*: 0.42; 95% CI: 0.08 and 0.76; *p* = 0.01; *I*^2^ = 35%; Figure 3). External focus also enhanced muscular endurance in the analysis for lower-body exercises (Cohen’s *d*: 0.95; 95% CI: 0.22 and 1.69; *p* = 0.01; *I*^2^ = 78%). There was no significant difference between external focus and control for upper-body exercises (Cohen’s *d*: 0.39; 95% CI: –0.02 and 0.80; *p* = 0.06; *I*^2^ = 39%).

**Figure 4:**
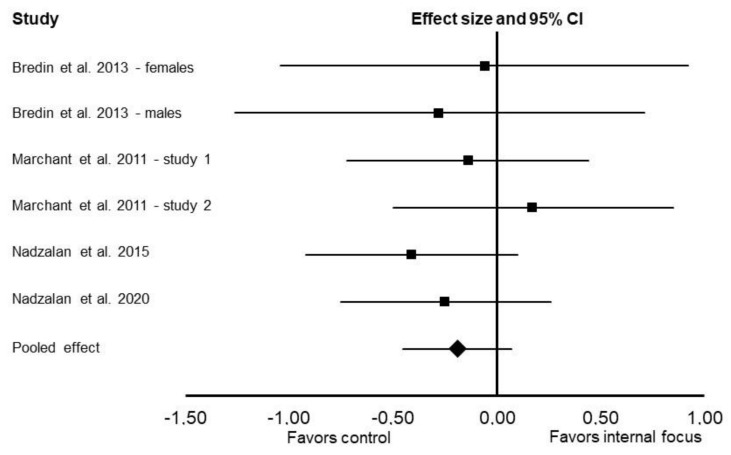
Results of the random-effects meta-analysis comparing the effects of internal focus vs. control on muscular endurance. Data are reported as effect sizes (Cohen’s *d*) and 95% confidence interval (CI). The diamond at the bottom presents the overall effect. The plotted squares denote effect sizes, and the whiskers denote their 95% CIs.

There was no significant difference between internal focus vs. control in the main meta-analysis (Cohen’s *d*: –0.19; 95% CI: –0.45 and 0.07; *p* = 0.14; *I*^2^ = 0%; Figure 4) and subgroup analyses for upper-body exercises (Cohen’s *d*: –0.15; 95% CI: –0.45 and 0.15; *p* = 0.32; *I*^2^ = 0%) and lower-body exercises (Cohen’s *d*: –0.33; 95% CI: –1.11 and 0.45; *p* = 0.41; *I*^2^ = 82%).

## 4. Discussion

The main finding of this review is that adopting an external focus of attention enhances muscular endurance. These performance-enhancing effects of external focus were found when comparing both with an internal focus and control condition. However, there was no significant difference between the internal focus and control conditions. Generally, these results remained consistent in subgroup analyses for upper-body vs. lower-body exercises. Overall, the results presented in this review suggest that individuals should use an external focus of attention for acute enhancement of muscular endurance.

The results presented herein add to the body of evidence supporting the positive effects of an external focus of attention on motor performance [1]. In one of the most cited reviews on the topic, Wulf summarized available studies and concluded that external focus of attention positively affects balance, accuracy, jumping, and performance in other sport-specific exercise outcomes [1]. At the time of that review, only one study [10] explored the effects of attentional focus strategies on muscular endurance, highlighting that the findings presented in this review are novel. It is generally accepted that the improvements in exercise performance with an external focus of attention are due to the constrained action hypothesis [1,19]. This hypothesis dictates that using an internal focus of attention leads the individual to focus only on one component of the movement [1,19]. However, movements in many exercise tasks are achieved by an integration of many muscles. Therefore, using an external focus of attention does not constrain the motor system, subsequently allowing for the task to be completed without omitting any of the contributors [1,19]. Studies have also demonstrated that an external focus of attention reduces antagonist muscle co-activation [20]. This finding is relevant as decreasing co-activation may increase force production, which could be associated with improvements in muscular endurance [21]. Additionally, studies have observed a reduced rating of perceived exertion (RPE) when performing the exercise tasks with an external focus of attention [22]. This should be considered when placed in the context of previous observations, which suggested that a reduction in RPE may enhance muscular endurance [23].

Several studies that evaluated the effects of external vs. internal focus on exercise performance also compared these two conditions to a control condition [10,24,25]. A benefit of external focus was found when compared to internal focus, but not when compared to the control condition [10,24,25]. Such an effect is explained by the findings that internal focus hampers exercise performance [1]. Therefore, when compared with the control, using an external focus of attention may not offer additional advantages in some populations (e.g., highly trained athletes) because the movements may already be highly automatized [24]. Even though most of the studies included in this review involved resistance-trained participants, a benefit of external focus was found when compared with an internal focus and the control condition. While this would suggest that resistance-trained participants may consider using an external focus of attention, future studies are needed to directly compare the effects of attentional focus strategies on muscular endurance between trained and untrained individuals. Wulf [1] suggested that the effects of attentional focus strategies are similar for novice, intermediate, and experienced performers, but more studies are needed to explore this topic with muscular endurance as the outcome. 

Besides the primary meta-analysis, subgroup analyses also explored the effects of internal vs. external focus vs. control on muscular endurance in lower-body vs. upper-body exercises. The results of these analyses generally mirrored those observed in the main analysis that considered all exercises. The pooled effects were higher for lower-body exercises (Cohen’s *d*: 0.95–1.36) than for upper-body exercises (Cohen’s *d*: 0.39–0.53). Generally, some lower-body exercises (e.g., squat) require more coordination than upper-body exercises (e.g., bench press) [14]. As external focus positively affects movement coordination, it may also have greater effects on muscular endurance in lower-body exercises [1,14]. Still, it should be considered that the 95% CIs of the analyses for lower-body vs. upper-body exercises were wide and overlapped. Perhaps even more importantly, it should be considered that all studies used complex, multi-joint exercises, such as the bench press, squat, and deadlift. This should be mentioned as complex movements require a greater level of multi-muscle and multi-joint coordination [26]. Therefore, the effects of an external focus of attention may be greater in multi-joint vs. single-joint resistance exercises [26]. To test this hypothesis, future studies are needed to compare the effects of external vs. internal focuses of attention on muscular endurance in various single-joint and multi-joint upper- and lower-body exercises.

Previous studies have reported that increases in muscular strength and muscle size with resistance training favour higher training volumes [27,28,29,30]. Given that the findings presented herein demonstrate an increase in muscular endurance and, therefore, training volume, it might be that using an external focus of attention over the long term also enhances outcomes such as muscular strength and hypertrophy. In one study, training with an external focus of attention increased 1RM strength in the squat and deadlift more than training with an internal focus of attention (13% vs. 9%) [31]. This finding aligns with the data reported in a recent meta-analysis on the topic [32]. However, the benefits of external focus should not be generalized to muscular hypertrophy, as one study reported that increases in elbow flexor thickness favoured training with an internal focus of attention [33]. Due to the paucity of studies on this topic, more research is still needed before making conclusive recommendations.

## 5. Conclusions

Compared with the internal focus of attention and control conditions, using an external focus of attention during resistance exercise acutely enhances muscular endurance. There was no significant difference between the internal focus of attention and control conditions. Generally, these results remained consistent in subgroup analyses for upper-body vs. lower-body exercises. From a practical perspective, the results presented in this review suggest that individuals should use an external focus of attention for acute enhancement of muscular endurance.

## Figures and Tables

**Table 1:** Summary of the studies included in the review. All studies explored the acute effects of attentional focus on muscular endurance.

Study	Participants	Participants Characteristics	External Focus Instructions	Internal Focus Instructions	Muscular Endurance Test ^a^	PEDro Score
Bredin et al. [8] *	16 young adults (8F/8M)	Age: 21 to 33 years; 1RM: n/a	Direct the concentration toward the floor as they completed each push-up	Concentrate on the arm muscles	Push-ups	6
Collum et al. [9] *	25 resistance-trained participants (9F/16M) ^b^	Age: 22 ± 2 years; 1RM: 76 ± 27 kg	“Drive the weight towards the ceiling”	“Drive the weight with your chest”	Bench press at 85% 1RM	6
Marchant et al. [10] – study 1 **	23 resistance-trained participants (7F/16M)	Age: 31 ± 12 years; 1RM: n/a	“Focus on moving and exerting force through and against the barbell”	“Focus on moving and exerting force with your arms”	Smith machine bench press with 40 kg (males) or 20 kg (females)	5
Marchant et al. [10] – study 2 **	17 resistance-trained participants (17M)	Age: 21 ± 1 years; Bench press 1RM: 95 ± 20 kg Squat 1RM: 184 ± 36 kg	Bench press: “focus on moving and exerting force through and against the barbell”Squat: “focus on moving and exerting force through and against the barbell”	Bench press: “focus on moving and exerting force with your arms” Squat: “focus on moving and exerting force with your legs”	Bench press and squat at 75% 1RM	5
Nadzalan et al. [11] *	30 resistance-trained participants (30M)	Age: 22 ± 1 years; Bench press 1RM: 75 ± 24 kg Deadlift 1RM: 143 ± 8 kg	Bench press: “focus on exerting force through and against the barbell” Deadlift: “focus your attention on pulling the bar up”	Bench press: “focus on exerting force with the arm” Deadlift: “focus your attention on extending your knees and hips”	Bench press and deadlift at 80% 1RM	6
Nadzalan et al. [12] **	30 resistance-trained participants (30M)	Age: 21 ± 1 years; Squat 1RM: 106 ± 13 kg Deadlift 1RM: 122 ± 13 kg	Squat: “focus on moving and exerting force through and against the barbell” Deadlift: “focus your attention on pulling the bar up”	Squat: “focus on moving and exerting force with your legs” Deadlift: “focus your attention on extending your knees and hips”	Squat and deadlift at 80% 1RM	5

1RM: one-repetition maximum; F: female; M: male; ^a^ all tests were performed to muscular failure; ^b^ only 23 participants completed all of the trials and were included in the meta-analysis; * study used a randomized crossover design; ** study used a non-randomized crossover design; n/a: not applicable.

## Data Availability

The data used for the meta-analysis are available from the corresponding author upon request.
